# Expression and Transcriptional Response of sirt1 in Yellow Catfish (Pelteobagrus fulvidraco) Under Acute Hypoxia Stress

**DOI:** 10.3390/ani16111679

**Published:** 2026-05-30

**Authors:** Tinglan Ku, Xinyuan Shan, Kai Zhang

**Affiliations:** 1 College of Marine Science and Engineering, Nanjing Normal University, Nanjing 210023, China; kutl_xx@126.com (T.K.); 19230226@njnu.edu.cn (X.S.); 2 College of Advanced Agricultural Sciences, Zhejiang Wanli University, Ningbo 315101, China

**Keywords:** yellow catfish (*Pelteobagrus fulvidraco*), *sirt1*, hypoxia stress, transcriptional response, intestinal injury, resveratrol

## Abstract

Hypoxia is a common stressor in aquaculture and severely compromises the physiological health of fish. The present study investigated the expression pattern and transcriptional response of the *sirt1* gene (*sirtuin1*) in yellow catfish (*Pelteobagrus fulvidraco*) under acute hypoxia stress. Sequence analysis of the yellow catfish *sirt1* gene indicated that the Sirt1 protein exhibits a high degree of sequence conservation among vertebrates. Tissue distribution analysis under normoxic conditions revealed that *sirt1* mRNA was most abundantly expressed in the brain and intestine of yellow catfish. Results from the hypoxia stress trial (dissolved oxygen 1.1 ± 0.2 mg/L) demonstrated that *sirt1* was the most hypoxia-responsive member of the *sirt* family, being significantly upregulated approximately 4.5-fold at 12 h. Subsequent functional validation experiments showed that *sirt1* expression was significantly decreased at 6 h of acute hypoxia; together with the marked upregulation at 12 h observed in the time-course experiment, this suggests that the response of *sirt1* to acute hypoxia is time-dependent. On this basis, a bidirectional intervention was performed using small interfering RNA (siRNA)-mediated knockdown of *sirt1* and resveratrol-mediated activation to analyze the effects of *sirt1* on downstream gene expression and intestinal histopathology. The results showed that inhibition of *sirt1* led to compensatory upregulation of *pdk1*, impairment of *ampkα* signaling and a further decrease in *stat3* expression, and aggravated hypoxia-induced intestinal histological damage, whereas resveratrol pretreatment partially restored the expression of the related genes and alleviated tissue injury. These findings indicate that *sirt1* is a transcriptionally responsive gene involved in the response of yellow catfish to hypoxia stress, and provide a reference for further investigation into hypoxia adaptation in yellow catfish.

## Simple Summary

Yellow catfish (*Pelteobagrus fulvidraco*) is an economically important freshwater fish in Chinese aquaculture that is highly susceptible to hypoxia in culture water, which can lead to mass mortality. We first examined the expression levels of the *sirt1* gene across multiple tissues of yellow catfish and found that it was most abundantly expressed in the brain and intestine, then investigated its transcriptional response in the intestine during hypoxia. We found that *sirt1* responds rapidly to hypoxic conditions and shows a dynamic transcriptional response associated with metabolic- and stress-related genes in fish. When *sirt1* expression was experimentally suppressed, intestinal tissue damage was markedly aggravated. Conversely, treatment with resveratrol partially restored downstream gene expression and alleviated tissue injury. These findings advance our understanding of how fish respond to hypoxic stress at the transcriptional level and may provide a basis for further investigation into hypoxia adaptation in aquaculture.

## 1. Introduction

Yellow catfish (*Pelteobagrus fulvidraco*) is an important freshwater economic fish species in China and is favored by consumers for its tender flesh and rich nutritional value [1]. According to FAO statistics, yellow catfish ranked 20th among freshwater aquaculture species worldwide in 2018, with aquaculture production almost entirely concentrated in China [2]. Its high-quality protein composition, low fat content, and favorable amino acid profile—including essential amino acids that meet WHO/FAO reference standards—render it nutritionally attractive not only to domestic consumers but also to markets in East and Southeast Asia, and it holds considerable potential for broader international consumption as aquaculture diversification continues globally [1,2]. In recent years, with the widespread adoption of intensive farming systems, the scale of yellow catfish aquaculture has continued to expand, becoming an important component of the freshwater aquaculture industry [2]. However, water-quality deterioration under high-density culture has become an increasingly prominent problem, and insufficient dissolved oxygen is one of the key environmental factors restricting the healthy farming of yellow catfish [3]. Under intensive culture conditions, high stocking density, heavy feed input and the accumulation of organic matter cause the oxygen consumption rate of the water body to frequently exceed its reoxygenation rate, leading to a sharp decline in dissolved oxygen [4,5]. Hypoxia stress can induce abnormal behavior, reduced feeding and stagnated growth in fish, and in severe cases may result in mass mortality [6,7]. In comparison with hypoxia-tolerant freshwater species such as crucian carp (*Carassius auratus*) and loach (*Misgurnus anguillicaudatus*), which possess robust anaerobic compensatory mechanisms and can sustain aerobic metabolism at very low dissolved oxygen concentrations, yellow catfish exhibits a comparatively limited anaerobic capacity and a relatively high critical oxygen threshold. Specifically, previous studies have shown that yellow catfish are relatively intolerant of hypoxia: observable stress responses—including elevated ventilation frequency, reduced swimming activity, suppressed feeding motivation, and activation of the cortisol-mediated stress axis—begin to appear when the dissolved oxygen drops below 3 mg/L, and when the dissolved oxygen falls below 1.5 mg/L the fish face a serious threat to survival [3,8]. Therefore, an in-depth investigation of the molecular mechanisms underlying the hypoxia response in yellow catfish is of considerable importance for improving culture survival rates. For yellow catfish, the intestine is an important organ of digestion and absorption and is also a target organ highly sensitive to environmental stress. Studies have shown that hypoxia stress can impair intestinal mucosal barrier function and increase permeability in fish, thereby inducing gut microbiota dysbiosis and secondary infection [9]. In addition, intestinal tissue has high metabolic activity and energy demand and is therefore sensitive to hypoxia-induced alterations in energy metabolism [10], which facilitates the functional validation of molecular mechanisms at the histomorphological level.

The sirtuin family is a group of evolutionarily conserved nicotinamide adenine dinucleotide (NAD^+^)-dependent protein deacetylases with homologs distributed across a wide taxonomic spectrum, from bacteria and yeast to invertebrates and vertebrates. In invertebrates, the founding *Sir2* gene and its orthologs have been extensively characterized in the nematode *Caenorhabditis elegans* (*sir-2.1*) and the fruit fly *Drosophila melanogaster* (*dSir2*), where Sir2-dependent deacetylase activity has been shown to mediate key aspects of caloric restriction responses, metabolic homeostasis, and lifespan regulation [11]. In humans, seven sirtuin paralogs (SIRT1–7) have been identified, with SIRT1 serving as the closest functional ortholog of yeast Sir2; human SIRT1 has been implicated in the regulation of metabolic syndrome, type 2 diabetes, cardiovascular disease, neurodegeneration, and cancer through deacetylation of a diverse array of transcription factors and co-regulators [12,13]. Sirt1 is the most extensively studied member of the family [14,15]; it has a wide range of substrates, including histones and numerous non-histone substrates such as p53, the forkhead box O (FOXO) family and peroxisome proliferator-activated receptor gamma coactivator 1-alpha (PGC-1α), and participates in the regulation of diverse biological processes such as cellular metabolism and stress responses through the deacetylation of these substrates [12]. The activity of Sirt1 depends on the intracellular level of NAD^+^, enabling it to directly sense the metabolic state of the cell [16]. In 2009, Cantó et al. revealed the coordinated regulatory mechanism between AMP-activated protein kinase (AMPK) and Sirt1, showing that AMPK activation increases intracellular NAD^+^ levels and thereby activates the deacetylase activity of Sirt1 [17]. With respect to the interaction between Sirt1 and hypoxia-inducible factor 1α (HIF-1α), multiple studies have demonstrated that Sirt1 can regulate the stability and transcriptional activity of HIF-1α through its deacetylation [18,19,20,21].

In fish, preliminary studies on the molecular characteristics and physiological functions of sirt1 have been conducted in several species, highlighting its important role in fish metabolic regulation and stress responses. In the model organism zebrafish (*Danio rerio*)—which belongs to the order Cypriniformes (family Danionidae) and thus represents a distinct teleost lineage from yellow catfish, which belongs to the order Siluriformes (family Bagridae); although both species belong to the superorder Ostariophysi, these two orders constitute separate major clades that diverged deep within the ostariophysan radiation [22]—the tissue expression profile of sirtuin family genes has been systematically characterized; *sirt1* mRNA showed the highest overall expression level among all examined organs, indicating its fundamental role in the regulation of tissue metabolism in fish [23]. A clustered regularly interspaced short palindromic repeats (CRISPR)-associated protein 9 (CRISPR-Cas9)-based knockout study of zebrafish *sirt1* further revealed its physiological importance: loss of *sirt1* led to a marked elevation in reactive oxygen species (ROS) levels, highly elevated expression of pro-inflammatory cytokines (*il-1b*, *il-6*, and *tnf-α*), intestinal atrophy, increased apoptosis and ultimately a shortened lifespan, confirming the key protective role of Sirt1 in maintaining oxidative homeostasis and inflammatory balance in fish [24]. Given the significant interordinal taxonomic distance between zebrafish and yellow catfish, the conservation of such sirt1-mediated functions across this phylogenetic gap would provide compelling evidence for the deep evolutionary conservation of this pathway among teleosts; however, whether analogous mechanisms operate in yellow catfish under stress conditions remains to be established. In largemouth bass (*Micropterus salmoides*), the *sirt1* gene has been successfully cloned and characterized; the encoded protein contains a highly conserved NAD^+^-binding domain and a zinc-finger binding module, is subcellularly localized to the nucleus and is predominantly expressed in the gonads, heart and liver. Dietary supplementation with resveratrol significantly increased Sirt1 protein levels and alleviated hepatic lipid deposition while enhancing antioxidant capacity through the protein kinase B (AKT)/FoxO1 signaling pathway, revealing the functional conservation of *sirt1* in lipid metabolism and oxidative stress regulation in teleosts [25]. In gilthead sea bream (*Sparus aurata*), *sirt1* is preferentially expressed in the brain, liver and white muscle and is dynamically regulated by nutritional status, and *sirt1* has been established as a highly responsive marker gene reflecting age- and season-dependent changes in energy metabolism [26]. By contrast, the molecular characteristics, tissue distribution and regulatory function of *sirt1* under hypoxia stress in yellow catfish have not yet been reported, which limits the understanding of its functional role in hypoxia adaptation.

Other members of the sirtuin family also play important roles in energy metabolism and stress responses [12]. Sirt2, the principal cytoplasmic sirtuin, is involved in the regulation of the cell cycle, adipocyte differentiation and inflammatory responses [27]. Sirt3, Sirt4 and Sirt5, as mitochondrial sirtuins, directly participate in the regulation of mitochondrial metabolism [22,28,29]. Sirt6 [30] and Sirt7 are predominantly localized to the nucleus and are involved in the transcriptional regulation of the genome [31]. A recent study in zebrafish showed that *sirt7* can suppress the expression of hypoxia-responsive genes by interacting with HIF-α subunits [32,33]. However, systematic studies of the sirtuin family in the hypoxia response of fish remain scarce, and research on important farmed species such as yellow catfish is particularly lacking.

On the basis of the above background, the present study used yellow catfish as the experimental subject and focused on the regulatory role of *sirt1* in the intestinal hypoxia response. First, bioinformatic analyses of the sequence features, physicochemical properties and evolutionary conservation of the yellow catfish *sirt1* gene were performed to characterize its molecular features. Next, tissue expression profiling under normoxia was used to determine the basal expression pattern of *sirt1* across the tissues of yellow catfish, and a hypoxia stress experiment was conducted to identify the *sirt* family member most responsive to hypoxia. A functional intervention model was then established to verify, at the transcriptional level, the regulatory effect of this member on the expression of downstream effector genes, combined with histopathological observation to assess its function in maintaining intestinal tissue homeostasis under hypoxic conditions. The present study aims to provide an initial investigation of the *sirt1*-mediated transcriptional response of the intestinal hypoxia response at the level of mRNA expression, offering a new perspective for a deeper understanding of hypoxia adaptation mechanisms in fish and providing a basis for further investigation for the healthy farming of yellow catfish and the development of stress-resistance strategies.

## 2. Materials and Methods

### 2.1. Experimental Materials

Healthy yellow catfish (*Pelteobagrus fulvidraco*) were obtained from the Lukou base of the Jiangsu Fisheries Research Institute (Nanjing, Jiangsu Province, China). The fish originated from a domesticated breeding population maintained at the institute and routinely used for aquaculture-related experimental studies. No specific commercial or genetically selected strain designation was available for this population. The fish were acclimatized for two weeks to the laboratory environment in plastic rearing tanks (80 cm × 60 cm × 50 cm) filled with dechlorinated tap water. During acclimation, the water temperature was maintained at 26 ± 0.5 °C, with the pH at 7.5 ± 0.1 and dissolved oxygen above 6.0 mg/L, with a 12L:12D photoperiod. The fish were fed twice daily at 9:00 and 18:00, each time at 1.5% of body weight, with commercial compound feed for yellow catfish. The health status of the fish was continuously monitored during acclimation. At the end of the acclimation period, individuals with a body weight of 6 ± 2 g, intact body surface and good vigor were selected for subsequent experiments. Throughout acclimation and the experiments, the water-quality parameters including the water temperature, pH and dissolved oxygen were monitored daily, and water was exchanged every two days to maintain good water quality.

Among the reagents used, TRIzol reagent was purchased from Invitrogen (Waltham, MA, USA); the PrimeScript RT reagent kit was purchased from TaKaRa Biotechnology (Dalian) Co., Ltd. (Dalian, China); Hieff qPCR SYBR Green Master Mix was purchased from Yeasen Biotechnology (Shanghai, China); and resveratrol (Res) was purchased from Shanghai Yuanye Bio-Technology Co., Ltd. (Yuanye Bio, Shanghai, China), with purity ≥ 99%. All other routine chemical reagents were of domestic analytical grade.

The instruments used included a Roche LightCycler 96 real-time quantitative PCR system (Roche, Basel, Switzerland), an Agilent 2100 Bioanalyzer (Agilent Technologies, Santa Clara, CA, USA) and a NanoPhotometer spectrophotometer (Thermo Scientific, Waltham, MA, USA), among others.

### 2.2. Experimental Design

**Figure 1:**
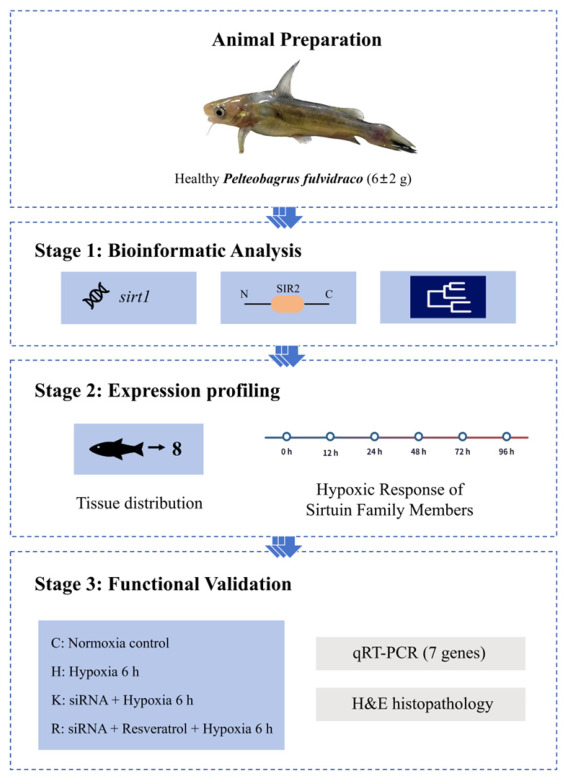
Schematic diagram of the experimental workflow. The study was conducted in three stages: (1) sequence characterization and phylogenetic analysis of *sirt1* in yellow catfish (*Pelteobagrus fulvidraco*); (2) expression profiling of the *sirt* family, including a normoxic tissue distribution assay (*n* = 3) and a hypoxic time-course assay *(n* = 3); and (3) functional validation using a four-group intervention model (C, H, K, and R; *n* = 10), with mRNA expression assessed by quantitative reverse-transcription PCR (qRT-PCR) and intestinal integrity evaluated by hematoxylin and eosin (H&E) staining. Hypoxia was established by nitrogen aeration to maintain dissolved oxygen at 1.1 ± 0.2 mg/L.

The overall experimental design of this study is shown in Figure 1. Before the experiments, the sequence information of the yellow catfish *sirt1* gene was first retrieved from the National Center for Biotechnology Information (NCBI) database, and protein domain prediction, multiple sequence alignment and phylogenetic analysis were performed to characterize its molecular features and evolutionary conservation; subsequent experiments were carried out on this basis. All animal experimental procedures were approved by the Institutional Animal Care and Use Committee (IACUC) of Nanjing Normal University (approval number: SYXK [Jiangsu] 2015-0028) and conducted in full compliance with China’s national Regulations on the Administration of Laboratory Animals (State Council, revised 2017; https://www.gov.cn/zhengce/content/2017-03/01/content\_5172146.htm, accessed on 22 May 2026). Details of the ethical review framework are provided in the Institutional Review Board Statement. All procedures strictly adhered to the principle of minimizing animal suffering. Prior to sample collection, the fish were deeply anesthetized with ethyl 3-aminobenzoate methanesulfonate (MS-222, 100 mg/L; Aladdin, Shanghai, China) until swimming responses ceased and opercular movements were markedly slowed, after which dissection and sampling were performed. At the end of the experiments, fish that were not used for sampling were humanely euthanized with an overdose of MS-222 (300 mg/L). Throughout the experiments, all personnel received standardized training, and sampling was conducted rapidly to minimize the duration of stress imposed on the fish.

### 2.3. Detection of sirt1 Tissue Distribution Under Normoxia

To determine the basal expression pattern of *sirt1* across different tissues of yellow catfish, healthy fish were randomly selected after acclimation under normoxic conditions (dissolved oxygen > 6.0 mg/L). The fish were anesthetized with MS-222 (100 mg/L) and rapidly dissected, and eight tissues—brain, intestine, heart, kidney, gill, skin, spleen and muscle—were collected, with three biological replicates per tissue. Samples were immediately snap-frozen in liquid nitrogen and then transferred to a −80 °C freezer for storage. The relative mRNA expression level of *sirt1* in each tissue was determined by quantitative reverse-transcription PCR (qRT-PCR).

### 2.4. Hypoxia Stress Experiment

To examine the expression changes of *sirt* family members and to screen for hypoxia-responsive candidate genes, a hypoxia stress experiment was first conducted. Six sampling time points (0, 12, 24, 48, 72 and 96 h) were set, with three biological replicates at each time point and three fish per replicate.

The hypoxic environment was established by nitrogen aeration. Fish were transferred to a sealed hypoxia-treatment tank, and high-purity nitrogen (purity > 99.99%) was introduced into the water to displace dissolved oxygen, keeping a stable concentration of dissolved oxygen at 1.1 ± 0.2 mg/L. Real-time monitoring was performed by using a portable dissolved-oxygen meter, and the nitrogen flow rate was dynamically adjusted to ensure the stability of the hypoxic conditions. Feeding was suspended during the hypoxic treatment, while other water-quality parameters such as water temperature and pH were kept constant.

Samples were collected at each preset time point. Before sampling, the fish were anesthetized with MS-222 (100 mg/L), after which the intestine was rapidly dissected, immediately snap-frozen in liquid nitrogen and subsequently stored at −80 °C. The relative mRNA expression levels of all seven family members *sirt1–7* in the intestine were measured by qRT-PCR to characterize their individual responses to hypoxia stress.

### 2.5. Functional Validation Experiment of sirt1

Based on the results of the time-course experiment, 6 h was selected as the acute hypoxia intervention time point, and small interfering RNA (siRNA) treatment was used to investigate the regulatory role of *sirt1*. This time point lies within the early dynamic stage of the *sirt1* response and thus facilitates the observation of the immediate effects of *sirt1* functional intervention on downstream signaling pathways. A similar strategy has been widely adopted in studies of the HIF-1α hypoxia response [34].

Although *sirt1* was detected across all eight examined tissues under normoxia, including the brain and heart (Section 2.3), the intestine was selected as the primary tissue for functional validation for three reasons: (1) *sirt1* expression in the intestine showed a pronounced and dynamic response during the hypoxia time course (Section 2.4); (2) the intestinal epithelium is among the most oxygen-sensitive tissues and constitutes a key site of hypoxia-induced structural injury in teleosts [9]; and (3) intestinal integrity could be assessed at both the molecular and morphological levels (qRT-PCR combined with H&E histopathology), enabling multi-dimensional evaluation of Sirt1 function. The roles of Sirt1 in other high-expression tissues such as the brain and heart under hypoxic conditions warrant further investigation in future studies.

siRNA design and screening: Three siRNAs (siRNA-1, siRNA-2 and siRNA-3) targeting different regions of the yellow catfish *sirt1* mRNA (GenBank accession no. NC_062521.1) were designed and synthesized by Sangon Biotech (Shanghai) Co., Ltd. (Shanghai, China). A negative-control siRNA (siRNA-NC) was included. To screen for the siRNA with the highest silencing efficiency, each siRNA was administered by intraperitoneal injection, and *sirt1* mRNA expression was measured under both normoxic and hypoxic conditions (dissolved oxygen 1.1 ± 0.2 mg/L, 6 h), with *n* = 4 per group. Based on the screening results, the siRNA with the highest silencing efficiency was selected for the subsequent functional validation experiment.

Four treatment groups were established. The normoxia control group (Group C) was reared under normal rearing conditions without any intervention. The hypoxia treatment group (Group H) was exposed to hypoxia at 1.1 ± 0.2 mg/L dissolved oxygen. The *sirt1* knockdown + hypoxia group (Group K) first received *sirt1* knockdown pretreatment: *sirt1* siRNA (50 μM, 10 μL per fish) was administered by intraperitoneal injection once every 3 days for a total of three injections, and hypoxia exposure was performed 24 h after the final injection. The *sirt1* knockdown + hypoxia + resveratrol rescue group (Group R) received the same RNA interference (RNAi) pretreatment as Group K and was then allowed to recover for one week, during which the fish were fed a functional diet supplemented with resveratrol, after which hypoxia exposure was performed. Samples were collected from each group after 6 h of hypoxia exposure, and the tissues sampled were the brain, intestine, heart, kidney, gill, skin, spleen and muscle. The one-week recovery period for Group R was set to allow sufficient time for the dietary resveratrol to be absorbed and to accumulate to biologically active concentrations in tissues [35]. We acknowledge that siRNA-mediated silencing is time-limited, typically persisting for several days to 1–2 weeks in vivo [36], and that the *sirt1* knockdown efficiency in Group R may have partially recovered by the time of hypoxia exposure. This partial recovery does not, however, invalidate the rescue comparison: in Group R, resveratrol is expected to activate residual or recovering Sirt1 activity, and the observed phenotypic differences from Group H reflect the net protective effect of Sirt1 functional restoration combined with resveratrol supplementation. This design limitation is explicitly acknowledged, and future studies employing stable knockdown strategies or pharmacological inhibitors with a defined duration would allow a cleaner dissociation of these effects. Each treatment group consisted of 10 fish, and each fish was sampled individually as an independent biological replicate (*n* = 10). Group K was designed to observe the effects of acute *sirt1* inhibition, whereas Group R was designed to observe the protective effects after functional restoration. The comparisons of these two groups with Group H thus reveal, respectively, the biological effects whereby Sirt1 deficiency aggravates injury and Sirt1 activation alleviates injury. A separate resveratrol-only hypoxia group (without siRNA pretreatment) was not included in the present study; this reflects a limitation of the current design, as such a group would have enabled the distinction between SIRT1-dependent and SIRT1-independent effects of resveratrol [37]. Future studies incorporating this control group would provide a more rigorous mechanistic dissection of resveratrol’s protective actions under hypoxia.

The sample collection method was the same as that of the hypoxia stress experiment. The relative mRNA expression levels of *sirt1* and its six key downstream regulatory genes (*ampkα*, *pgk1*, *pdk1*, *epoa*, *stat3*, and *mapk1*) were determined by qRT-PCR to systematically evaluate the regulatory effects of Sirt1 functional status on the intestinal hypoxia signaling pathway.

### 2.6. Analytical Methods

#### 2.6.1. Bioinformatic Analysis of the *sirt1* Gene

The cDNA sequence (accession no. XM_027165852.2) and the encoded protein sequence (accession no. XP_027021653.2) of the yellow catfish *sirt1* gene were retrieved from the NCBI GenBank database. The ExPASy ProtParam tool (https://web.expasy.org/protparam/, accessed on 15 April 2026) was used to predict the physicochemical parameters of the Sirt1 protein, including the molecular weight, theoretical isoelectric point, amino acid composition, instability index, aliphatic index and grand average of hydropathicity. Subcellular localization was predicted using WoLF PSORT (https://wolfpsort.hgc.jp/, accessed on 15 April 2026). Functional domains of the Sirt1 protein were identified using the NCBI Conserved Domain Database (CDD, https://www.ncbi.nlm.nih.gov/cdd/, accessed on 20 April 2026). The schematic representation of the protein domains was generated using the IBS tool (http://ibs.biocuckoo.org/, accessed on 20 April 2026).

Sirt1 homologous protein sequences from nine representative vertebrate species were retrieved from the NCBI database, including channel catfish (*Ictalurus punctatus*, XP_017317358.1), zebrafish (*Danio rerio*, XP_001334440.5), largemouth bass (*Micropterus salmoides*, XP_038592740.1), Nile tilapia (*Oreochromis niloticus*, XP_005473903.1), large yellow croaker (*Larimichthys crocea*, XP_010732868.1), Atlantic salmon (*Salmo salar*, XP_014054654.1), African clawed frog (*Xenopus laevis*, NP_001091195.1), mouse (*Mus musculus*, NP_062786.1) and human (*Homo sapiens*, NP_036370.2). Full-length multiple sequence alignment was then performed using Clustal Omega v1.2.4 (https://www.ebi.ac.uk/jdispatcher/msa/clustalo, accessed on 20 April 2026), from which the amino acid sequence identities between species were obtained from the resulting Percent Identity Matrix. Multiple sequence alignment was also carried out using the ClustalW algorithm in MEGA12 software [38], and the alignment results were visualized with ESPript 3.0 (https://espript.ibcp.fr/, accessed on 21 April 2026). Based on the aligned amino acid sequences, a phylogenetic tree was constructed using the Neighbor-Joining (NJ) method [39]; evolutionary distances were calculated with the Jones–Taylor–Thornton (JTT) matrix-based method [40], rate variation among sites was modelled with a gamma distribution (shape parameter = 1.00), and node reliability was assessed with 1000 bootstrap replicates [41]. The analysis was performed in MEGA12 software [38].

#### 2.6.2. qRT-PCR Validation

Total RNA was extracted from intestinal tissue using the TRIzol method. RNA concentration and purity were measured with a NanoPhotometer spectrophotometer (Thermo Scientific, USA), and RNA integrity was assessed by 1.0% agarose gel electrophoresis to ensure the absence of evident degradation. Reverse transcription was then performed with the PrimeScript RT reagent kit (Takara, Hong Kong, China) to synthesize cDNA, which was stored at −20 °C until use.

Specific primers for the target and endogenous reference genes were designed based on the sequence information from NCBI GenBank and were synthesized by Sangon Biotech (Shanghai) Co., Ltd. The PCR products were verified by sequencing, with the sequence identity above 99%. The primer sequences for each gene are provided in the Supplementary Material Table S2.

qRT-PCR analysis was performed with Hieff^®^ qPCR SYBR^®^ Green (Yeasen Biotechnology, Shanghai, China) on a Roche LightCycler 96 system (Rotkreuz, Switzerland). Each sample was run in three technical replicates, with *β-actin* as the endogenous reference gene, and the relative expression levels of the target genes were calculated using the 2^−∆∆Ct^ method. The calibrator group for the tissue distribution experiment was the tissue with the lowest expression; the calibrator group for the hypoxia stress experiment was the 0 h group; and the calibrator group for the functional validation experiment was Group C.

#### 2.6.3. Intestinal Histopathological Examination

To evaluate, at the histomorphological level, the effects of the *sirt1* functional status on the structural integrity of the intestine under hypoxic conditions, midgut tissues from each treatment group of the *sirt1* functional validation experiment were subjected to histopathological examination. Midgut segments approximately 0.5 cm in length were gently rinsed with physiological saline and then fixed in 4% paraformaldehyde solution for more than 24 h. The tissues were embedded in paraffin, sectioned and stained with hematoxylin and eosin (H&E); after mounting, morphological observation and image acquisition were performed under a light microscope. Images of the stained sections were observed and captured using SlideViewer 2.9 software under a 400× objective; scale bar = 50 μm. The observation indicators included the integrity of the intestinal mucosal epithelium, the morphology of the villus structure, the cellular density of the lamina propria–submucosa and the arrangement of muscularis fibers, and were used to comprehensively evaluate the pathological changes in the intestine under different treatment conditions. As in most teleosts, yellow catfish lack a distinct muscularis mucosae, and there is no clear histological demarcation between the lamina propria and the submucosa; the two form a continuous connective tissue region. Accordingly, this region is uniformly described in the present paper as the lamina propria–submucosa.

In addition, the histopathological changes in each group were semi-quantitatively scored. The scoring criteria were adapted from the semi-quantitative histopathology scoring method for fish established by Baums et al. [42], with modifications based on the histological features of the yellow catfish intestine. Evaluation was carried out on four aspects: epithelial integrity, villus structure, lamina propria–submucosa inflammation and muscularis structure. Each aspect was scored on a scale of 0–3, where 0 indicates normal, 1 mild injury, 2 moderate injury and 3 severe injury. The individual scores were summed to yield a total score ranging from 0 to 12. The scoring was performed by a rater experienced in fish histopathology, who conducted two independent scorings; the mean of the two scorings was taken as the final result. The scores are not intended to serve as a basis for statistical analysis, but rather as a supplementary semi-quantitative reference for the morphological description. The detailed Semi-quantitative Histopathological Scoring Criteria for Intestinal Tissue are provided in the Supplementary Material Table S1.

### 2.7. Data Statistics and Analysis

All data are expressed as mean ± standard deviation (mean ± SD), with at least three biological replicates per group and three technical replicates per sample. Data were analyzed with SPSS 26.0 (IBM, Armonk, NY, USA). The data were first subjected to a normality test (Shapiro–Wilk test) and a homogeneity-of-variance test (Levene’s test). Data that conformed to a normal distribution and showed homogeneity of variance were analyzed by one-way analysis of variance (one-way ANOVA), with the least significant difference (LSD) test used for post hoc pairwise comparisons; data with unequal variances were analyzed by Welch’s ANOVA with the Games–Howell post hoc test for pairwise comparisons. Different letters denote statistically significant differences between groups (*p* < 0.05). Figures were generated with GraphPad Prism 9.5.

## 3. Results

### 3.1. Sequence Features and Evolutionary Analysis of the Yellow Catfish sirt1 Gene

The cDNA of the yellow catfish *sirt1* gene (GenBank accession no. XM_027165852.2) is 2581 bp in length and encodes a protein of 726 amino acid residues (GenBank accession no. XP_027021653.2). ProtParam prediction of the physicochemical properties showed that the protein has a molecular weight of 80.67 kDa and a theoretical isoelectric point (pI) of 4.78, indicating that it is an acidic protein. In terms of amino acid composition, the total number of negatively charged residues such as aspartic acid (Asp) and glutamic acid (Glu) was 118, substantially greater than the 73 positively charged residues such as arginine (Arg) and lysine (Lys), which is consistent with the acidic pI. The instability index was 51.23 (greater than 40), predicting the protein to be unstable. The aliphatic index was 72.20, and the grand average of hydropathicity (GRAVY) was −0.599, suggesting that the protein is overall hydrophilic. WoLF PSORT subcellular-localization prediction indicated that the yellow catfish Sirt1 protein is most likely localized to the cytoplasm and, secondarily, to the nucleus (WoLF PSORT scores of 19 and 5.5, respectively), which is similar to the subcellular-localization characteristic of mammalian Sirt1, which mainly shuttles between the nucleus and the cytoplasm [12].

**Figure 2:**
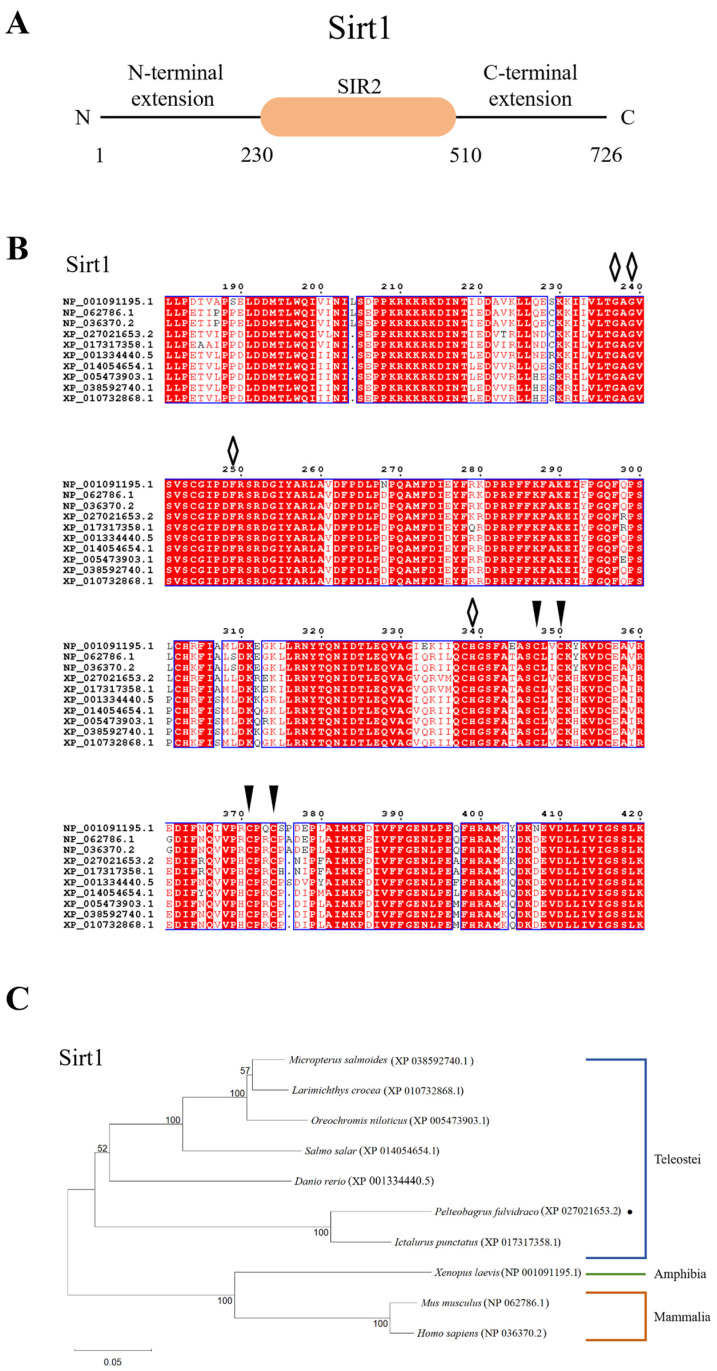
Structural characterization and evolutionary analysis of the Sirt1 protein in yellow catfish (*Pelteobagrus fulvidraco*). (**A**) Schematic representation of the domain architecture of the Sirt1 protein in yellow catfish. The full-length protein comprises 726 amino acids, including an N-terminal extension (residues 1–229), a conserved SIR2 superfamily catalytic domain (residues 230–510) and a C-terminal extension (residues 511–726). Domain information was obtained from the NCBI Conserved Domain Database (CDD), and the schematic was drawn with the IBS tool. (**B**) Partial multiple sequence alignment of yellow catfish Sirt1 against homologous proteins from nine representative vertebrate species. A red background denotes strictly conserved residues, whereas red lettering indicates highly conserved residues. Diamonds (◇) mark key residues of the NAD^+^-binding site, and inverted triangles mark key residues of the Zn^2+^-binding motif. The alignment was generated with the ClustalW algorithm implemented in MEGA12 and visualized with ESPript 3.0; the complete alignment is shown in Supplementary Figure S1. (**C**) Phylogenetic tree based on Sirt1 amino acid sequences. Multiple sequence alignment was performed using MUSCLE implemented in MEGA12 with default parameters. The tree was constructed using the Neighbor-Joining (NJ) method. Evolutionary distances were calculated with the Jones–Taylor–Thornton (JTT) matrix-based method, and among-site rate variation was corrected using a Gamma distribution model (shape parameter = 1.00). Bootstrap support values (%) from 1000 replicates are shown at nodes (values > 50% are displayed). The scale bar represents 0.05 amino acid substitutions per site. The black dot (●) denotes the yellow catfish (*Pelteobagrus fulvidraco*) analysis.

NCBI Conserved Domain Database (CDD) analysis showed that the yellow catfish Sirt1 protein contains a conserved catalytic domain of the SIR2 superfamily (Sir2 superfamily domain) spanning amino acid residues 230–510 (Figure 2A). This catalytic domain is the core region through which sirtuin family proteins exert their NAD^+^-dependent deacetylase activity [14,15]. The catalytic domain is flanked by an N-terminal extension (residues 1–229) and a C-terminal extension (residues 511–726). These two regions are less conserved among species and are thought to be involved in regulating the subcellular localization, protein–protein interactions and post-translational modifications of the Sirt1 protein [43].

To assess the evolutionary conservation of the yellow catfish Sirt1 protein, its amino acid sequence was subjected to multiple sequence alignment with Sirt1 homologues from nine representative species (Figure 2B). The results showed that the SIR2 catalytic domain was highly conserved among all species examined, and the key residues of the NAD^+^-binding site and the Zn^2+^-binding motif were completely conserved across all species (Figure 2B; the full alignment is shown in Supplementary Figure S1), indicating that the catalytic function of the Sirt1 protein is highly conserved among vertebrates. Full-length sequence alignment showed that the amino acid identity between yellow catfish Sirt1 and channel catfish was the highest at 80.36%, while that with zebrafish was 57.91%; identities with largemouth bass, Nile tilapia and large yellow croaker were 60.87%, 60.06% and 60.56%, respectively; that with Atlantic salmon was 59.23%; that with *Xenopus* was 53.43%; and those with mouse and human were 55.71% and 54.92%, respectively. These results indicate that the Sirt1 protein exhibits a relatively high degree of sequence conservation among vertebrates, with an especially high level of conservation in the catalytic domain, whereas the N- and C-terminal extensions are more variable, consistent with previous reports in the literature [43,44].

The phylogenetic tree constructed from the Sirt1 amino acid sequences of 10 species (Figure 2C) showed that yellow catfish Sirt1 clustered with channel catfish (*Ictalurus punctatus*) with 100% bootstrap support, forming the Siluriformes clade. Channel catfish is the most commercially important freshwater aquaculture species in the United States and has been widely introduced into aquaculture systems across China and Southeast Asia; it additionally serves as a primary model organism in catfish genomics and comparative molecular biology [45]. Within teleosts, largemouth bass and large yellow croaker clustered first (57% bootstrap support) and then grouped with Nile tilapia to form the Eupercaria clade (100% bootstrap support); the Eupercaria clade clustered with Atlantic salmon (Salmoniformes) (52% bootstrap support) and subsequently with zebrafish (Cypriniformes). The Siluriformes clade (yellow catfish and channel catfish) occupied a position relatively independent of the above groups within the teleost branch. The amphibian (African clawed frog) and the mammals (mouse and human, with 100% bootstrap support between them) together formed the tetrapod outgroup with 100% bootstrap support. The overall topology broadly reflected the phylogenetic relationships of vertebrates [22], further confirming the evolutionary conservation of the Sirt1 protein. The remarkable retention of the SIR2 catalytic core across taxa as phylogenetically distant as teleosts and mammals is consistent with Jacob’s concept of evolution as a process of “tinkering” (*bricolage*) [46], whereby natural selection conserves and repurposes pre-existing functional modules rather than constructing entirely novel machinery—the invariant catalytic domain of Sirt1 exemplifies precisely such a retained molecular tool. It should be noted that phylogenetic trees constructed from a single gene may show some discrepancies with species phylogenies inferred from whole-genome data, which is a common phenomenon in protein evolutionary analysis. The evolutionary analysis was performed using the Neighbor-Joining method [39]; evolutionary distances were calculated with the JTT matrix-based method [40]; rate variation among sites was corrected using a gamma distribution model (shape parameter = 1.00); 1000 bootstrap replicates were performed [41]; and the analysis was carried out in MEGA12 software [38].

### 3.2. Normoxic Expression Profile of sirt1 in Different Tissues of Yellow Catfish

**Figure 3:**
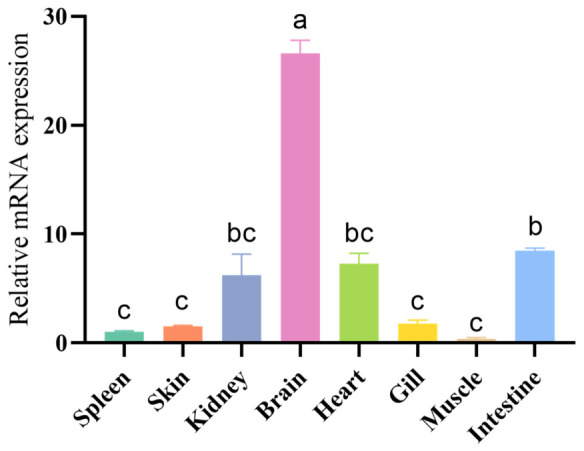
Normoxic expression profile of *sirt1* in different tissues of yellow catfish. The relative mRNA expression levels of *sirt1* in the brain, intestine, heart, kidney, gill, skin, spleen and muscle of yellow catfish under normoxic conditions were determined by qRT-PCR. *β-actin* was used as the endogenous reference gene, and the expression was calculated using the 2^−ΔΔCt^ method with muscle tissue used as the calibrator for normalization. Data are presented as mean ± standard deviation (SD) (*n* = 3). Different letters indicate significant differences among groups (*p* < 0.05, one-way analysis of variance (ANOVA) or Welch’s ANOVA, followed by the least significant difference (LSD) or Games–Howell post hoc test).

The *sirt1* gene was expressed in all examined tissues of yellow catfish, but the expression levels differed markedly among tissues. As shown in Figure 3, *sirt1* expression was highest in the brain and significantly exceeded that of all other tissues (*p* < 0.05). Expression in the intestine (8.62 ± 0.39) ranked second and was significantly higher than in the heart (7.36 ± 1.72) and kidney (6.32 ± 3.40) (*p* < 0.05). No significant difference was observed between the heart and kidney (*p* > 0.05), but both were significantly higher than the gill (1.78 ± 0.58), skin (1.53 ± 0.18), spleen (1.01 ± 0.20) and muscle (0.40 ± 0.15) (*p* < 0.05). The lowest expression was detected in muscle. Overall, *sirt1* mRNA showed relatively high basal expression in metabolically active tissues with high energy demand, such as the brain, intestine and heart, whereas expression was comparatively low in the muscle, skin and other tissues.

### 3.3. Intestinal Expression Patterns of the sirt Family Under Hypoxic Stress

**Figure 4:**
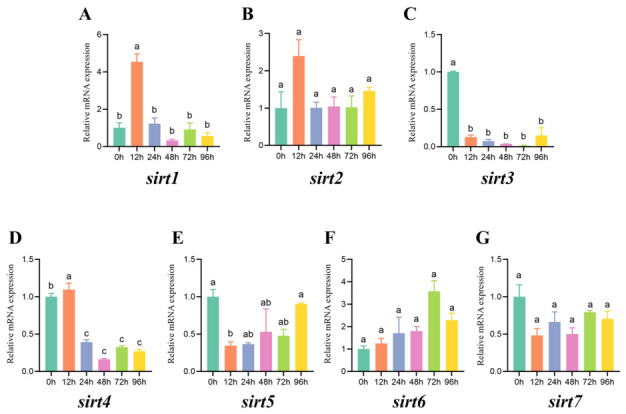
Time course of mRNA expression of *sirt* family members in the intestine of yellow catfish under hypoxic stress. Yellow catfish were exposed to hypoxia (dissolved oxygen 1.1 ± 0.2 mg/L) for 0, 12, 24, 48, 72 and 96 h, and the relative mRNA expression levels of *sirt1* (**A**), *sirt2* (**B**), *sirt3* (**C**), *sirt4* (**D**), *sirt5* (**E**), *sirt6* (**F**) and *sirt7* (**G**) in the intestine were measured by qRT-PCR. *β-actin* was used as the endogenous reference gene, and expression was calculated with the 2^−∆∆Ct^ method using the 0 h group as the calibrator. Data are presented as mean ± SD (*n* = 3 biological replicates). Different letters indicate significant differences among groups (*p* < 0.05, one-way ANOVA or Welch’s ANOVA selected based on Levene’s test for homogeneity of variance, followed by the LSD or Games–Howell post hoc test, respectively).

To identify the members of the *sirt* family that are most responsive to hypoxia, qRT-PCR was used to monitor changes in the mRNA expression of *sirt1–7* in the intestine of yellow catfish at different time points of hypoxic exposure (0, 12, 24, 48, 72 and 96 h). The results are shown in Figure 4.

Individual members of the *sirt* family displayed clearly distinct response patterns to hypoxic stress. *sirt1* exhibited the typical features of an acute stress response: its expression was significantly elevated to approximately 4.5-fold that of the control at 12 h of hypoxia (*p* < 0.05), after which it declined rapidly and returned to basal levels by 24 h. It should be noted that the present time-course experiment did not include a 6 h time point; data from the independent acute hypoxia intervention in Section 3.5 indicate that *sirt1* was markedly suppressed at 6 h of hypoxia. This apparent discrepancy is consistent with the conflicting reports in the literature: hypoxia has been shown to suppress *sirt1* transcription via a C-terminal-binding protein (CtBP)-dependent mechanism linked to reduced NAD^+^/NADH [47], while separately, HIF-1α has been reported to transcriptionally activate the *sirt1* promoter via hypoxia-response elements, driving upregulation at later time points [48]. Whether these two regulatory mechanisms operate sequentially in the yellow catfish intestine—with early suppression followed by HIF-driven recovery—warrants confirmation by a denser time-course study encompassing intermediate time points such as 0, 3, 6, 9, and 12 h. *sirt2* showed a similar early-upregulation trend, reaching its peak at 12 h (about 2.3-fold the control), but the inter-group difference did not reach statistical significance.

In contrast to these early-response patterns, *sirt3* and *sirt4* showed sustained downregulation. The expression of *sirt3* was significantly reduced to approximately 10% of the control level as early as 12 h after hypoxic exposure (*p* < 0.05) and remained low throughout the experiment, whereas *sirt4* expression decreased by approximately 60–90% after 24 h (*p* < 0.05).

In addition, *sirt6* exhibited a delayed-upregulation pattern: its expression remained largely unchanged during the early phase of hypoxia (12–48 h) and showed an upward tendency at 72 h. *sirt5* and *sirt7* fluctuated only slightly throughout the hypoxic treatment, and no time point showed a statistically significant difference from the control (*p* > 0.05).

Taken together, *sirt1* was the most pronounced and most rapidly responsive member of the *sirt* family in the intestinal hypoxic response of yellow catfish under the present experimental conditions, and was therefore selected as the target gene for the subsequent functional validation experiments.

### 3.4. Screening and Validation of siRNAs Targeting sirt1

**Figure 5:**
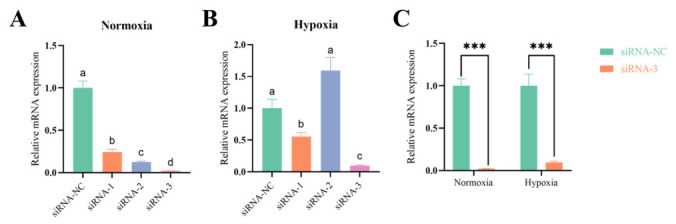
Screening of *sirt1* small interfering RNAs (siRNAs)s and validation of silencing efficiency. Three siRNAs (siRNA-1, siRNA-2, and siRNA-3) targeting yellow catfish *sirt1* mRNA were designed and administered by intraperitoneal injection. *sirt1* mRNA expression was then measured under normoxia (**A**) and hypoxia ((**B**), dissolved oxygen 1.1 ± 0.2 mg/L, 6 h). During the experiment, siRNA-NC was used as the negative control siRNA, and *β-actin* as the endogenous reference gene. Data are presented as mean ± SD (*n* = 4). Based on the screening results, siRNA-3 was selected for subsequent functional validation experiments. In panels (**A**,**B**), different letters indicate significant differences among groups (*p* < 0.05, one-way ANOVA or Welch’s ANOVA, followed by the LSD or Games–Howell post hoc test). In panel (**C**), *** indicates an extremely significant difference between groups (*p* < 0.001, Student’s *t*-test).

To determine the optimal siRNA for the functional validation experiments, the silencing efficiencies of three siRNAs targeting *sirt1* were compared under normoxic and hypoxic conditions (Figure 5). Under normoxia (Figure 5A), siRNA-1, siRNA-2 and siRNA-3 all reduced *sirt1* mRNA expression to various extents, with siRNA-3 producing the highest knockdown efficiency. Under hypoxia (Figure 5B), siRNA-3 likewise exhibited the strongest silencing effect. These results confirmed that siRNA-3 significantly decreased *sirt1* mRNA expression under both normoxic and hypoxic conditions (*p* < 0.001) (Figure 5C); siRNA-3 was therefore selected for all subsequent functional validation experiments.

### 3.5. Expression Changes of Key Genes Under Acute Hypoxia

**Figure 6:**
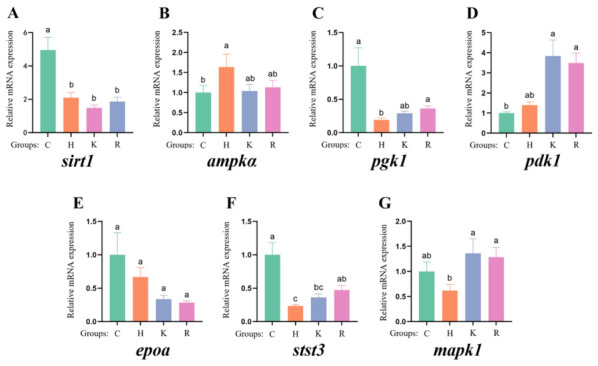
Changes in the mRNA expression of key genes in the intestine of yellow catfish under acute hypoxia (dissolved oxygen 1.1 ± 0.2 mg/L) for 6 h. Four experimental groups were established: normoxic control (group C), hypoxic treatment (group H), *sirt1* siRNA + hypoxia (group K), and *sirt1* siRNA + hypoxia + resveratrol rescue (group R). The relative mRNA expression levels of *sirt1* (**A**), *ampkα* (**B**), *pgk1* (**C**), *pdk1* (**D**), *epoa* (**E**), *stat3* (**F**) and *mapk1* (**G**) were measured by qRT-PCR. *β-actin* served as the endogenous reference gene, and expression was calculated with the 2^−∆∆Ct^ method, using the normoxic control group (group C) as the calibrator for normalization. Data are presented as mean ± SD (*n* = 10). Different letters indicate significant differences among groups (*p* < 0.05, one-way ANOVA or Welch’s ANOVA selected based on Levene’s test for homogeneity of variance, followed by the LSD or Games–Howell post hoc test, respectively).

To verify the causal regulatory role of *sirt1* in the acute hypoxic response, an acute hypoxia intervention model was established comprising four treatment groups: normoxic control (group C), hypoxic treatment (group H), *sirt1* knockdown + hypoxia (group K), and *sirt1* knockdown + hypoxia + resveratrol rescue (group R). On the basis of the siRNA screening results (Figure 5), siRNA-3 was used in group K to silence *sirt1*. A hypoxic exposure of 6 h was selected as the intervention time point, consistent with established protocols for acute hypoxia studies in yellow catfish [3] and to capture early-phase transcriptional responses prior to any potential adaptive recovery, and the relative mRNA expression levels of *sirt1* and six related genes (*ampkα*, *pgk1*, *pdk1*, *epoa*, *stat3*, and *mapk1*) were measured. The results are shown in Figure 6.

In group H, *sirt1* expression was significantly reduced by 58.0% relative to group C (*p* < 0.05), indicating that 6 h of acute hypoxia markedly suppressed *sirt1* transcription in the intestine (Figure 6A). In group K, *sirt1* expression was 72.0% lower than in group C (*p* < 0.05), a further decrease compared with group H, indicating that siRNA treatment produced an additive suppressive effect under hypoxic conditions. In group R, *sirt1* expression was 28.6% higher than in group K, recovering to 36% of the level in group C and thereby showing a partial restoration, although it did not return completely to control levels.

Among the energy-metabolism-related genes (Figure 6B–D), *ampkα* expression in group H was significantly upregulated to approximately 1.6-fold that of the control (*p* < 0.05), indicating that acute hypoxia activated the AMPK signaling pathway; in group K, *ampkα* expression fell back to a level close to that of the control, whereas group R maintained a relatively high level. *pdk1* expression in group H increased to approximately 1.4-fold, but the difference did not reach statistical significance; in group K, *pdk1* expression rose significantly to approximately 3.8-fold (*p* < 0.05), the largest change observed among all genes, and remained at approximately 3.5-fold in group R (*p* < 0.05). In contrast to the pattern of *pdk1*, *pgk1* was already significantly downregulated in group H to approximately 0.2-fold (*p* < 0.05); although a slight rebound occurred in groups K and R, the expression remained below the control level. Collectively, these changes in the three energy-metabolism genes revealed a noteworthy phenomenon: in group K, where *sirt1* was suppressed, *ampkα* signaling was attenuated while *pdk1* was sharply upregulated as a compensatory response, suggesting that a reduction in *sirt1* expression may disturb the coordinated expression of genes involved in energy sensing and metabolic regulation.

Among the genes related to signal transduction and oxygen-carrying capacity (Figure 6E–G), *epoa*, a classical HIF target gene, tended to be downregulated in all treatment groups, but none of the differences reached statistical significance (*p* > 0.05). *stat3* expression in group H was significantly reduced to approximately 0.22-fold that of the control (*p* < 0.05); it rebounded slightly in group K (approximately 0.35-fold, *p* < 0.05) and recovered to approximately 0.5-fold in group R, showing an overall pattern of hypoxia-induced suppression with partial alleviation by resveratrol. *mapk1* was significantly downregulated to approximately 0.6-fold in group H (*p* < 0.05), but was conversely upregulated in groups K and R (both approximately 1.3-fold, *p* < 0.05 vs. group H), displaying a response pattern entirely different from that of the other downregulated genes. In contrast to other downregulated genes, *mapk1* showed an inverse relationship with the *sirt1* expression level across groups, being downregulated in group H but upregulated in groups K and R.

Taken together, the expression profiles of the four treatment groups showed clearly differentiated features. Group H was characterized by upregulation of *ampkα* together with downregulation of *sirt1*, *pgk1*, *stat3* and *mapk1*, reflecting the immediate stress state induced by acute hypoxia. Group K was dominated by a pronounced compensatory upregulation of *pdk1* and a return of *ampkα* toward the baseline, suggesting that the downregulation of *sirt1* was accompanied by altered expression patterns of hypoxia-related metabolic genes. In group R, sustained high expression of *pdk1* coexisted with partial recovery of *pgk1* and *stat3*, indicating that resveratrol exerts a certain corrective effect on the expression of specific genes. These inter-group differences provide the experimental basis for the discussion of the regulatory mechanism of *sirt1* in the Discussion.

### 3.6. Histopathological Observations

**Figure 7:**
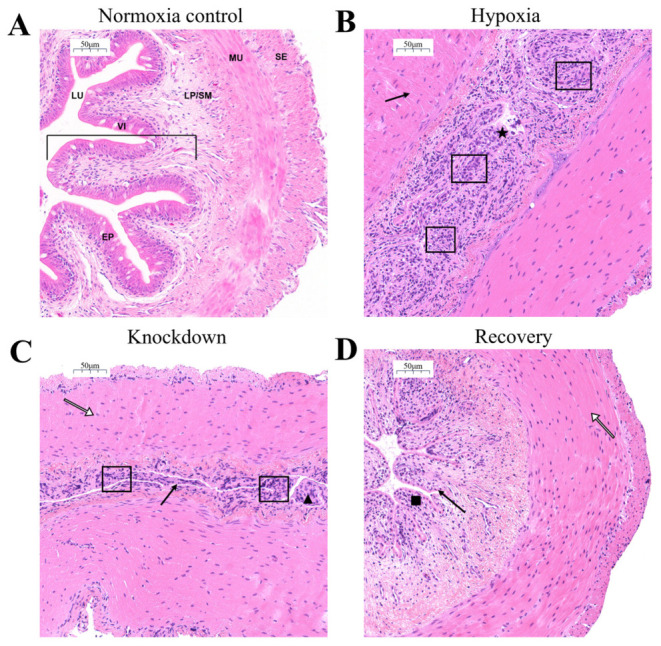
H&E-stained histopathological sections of the midgut of yellow catfish in each treatment group. Midgut tissues of yellow catfish from each group were fixed in 4% paraformaldehyde, embedded in paraffin, and stained with hematoxylin and eosin (H&E). (**A**): Normoxic control group (group C); (**B**): hypoxic treatment group (group H); (**C**): *sirt1* siRNA + hypoxia group (group K); (**D**): *sirt1* siRNA + hypoxia + resveratrol rescue group (group R). Scale bar = 50 μm. SE, serosa; MU, muscle layer; LP/SM, lamina propria–submucosa; VI, villi; EP, mucosal epithelium; LU, intestinal lumen. In group H, slight edema is visible at the villus tips (★), and the boxed area shows aggregations of dark-purple dots, indicating increased inflammatory cell infiltration. In group K, villi are atrophied and shortened (▲), the epithelial layer is thinned and loosely arranged (arrows), inflammatory infiltration is markedly increased in the boxed area, and the muscle-layer fibers are disorganized (open arrows). In group R, the villus structure is markedly improved compared with group K, with restored tip morphology (■), largely recovered epithelium (arrows), and a partially restored muscle layer (open arrows).

To assess the impact of the *sirt1* functional status on the intestinal structure under hypoxia, midgut tissues from each treatment group were examined by H&E staining. The results are shown in Figure 7.

In group C, the intestinal structure was intact: the mucosal epithelial cells were arranged in a columnar pattern, the villus structure was well defined, and the submucosa and muscle layer showed regular organization, with no obvious pathological changes. In group H, slight edema was observed at the villus tips, and an increased tendency for inflammatory cell infiltration was evident in the lamina propria–submucosa; these morphological alterations are consistent with the damaging effects of acute hypoxia on intestinal tissue. Group K showed more pronounced tissue injury, including thinning of the mucosal epithelium, villus atrophy and disruption, increased inflammatory infiltration in the lamina propria–submucosa, and disordered arrangement of muscle-layer fibers, suggesting that suppression of *sirt1* function may aggravate hypoxia-induced injury. The intestinal morphology of group R was markedly improved compared with group K: the mucosal epithelium had largely recovered, the villus structure was relatively intact with restored morphology, and inflammatory infiltration in the lamina propria–submucosa was attenuated—observations consistent with the tissue-protective effect of resveratrol [37].

**Figure 8:**
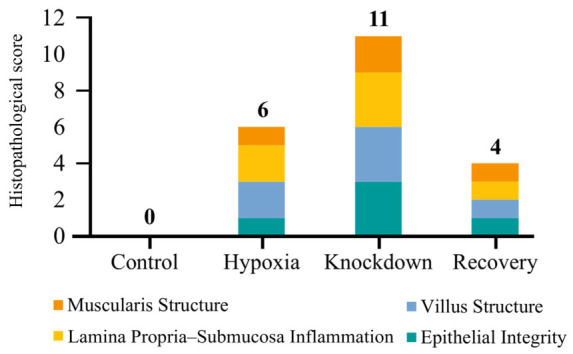
Stacked bar chart of semi-quantitative histopathological scores of midgut tissues in each treatment group of yellow catfish. Based on the semi-quantitative scoring data in Table 1, a stacked bar chart was used to illustrate the degree of tissue injury in each treatment group. Different colors represent the four scoring dimensions: epithelial integrity, villus architecture, lamina propria–submucosa inflammation, and muscle-layer structure. The numbers on top of each bar indicate the total score of the respective group. C, normoxic control group; H, hypoxic treatment group; K, *sirt1* siRNA + hypoxia group; R, *sirt1* siRNA + hypoxia + resveratrol rescue group. With *n* = 1 per group, the scores represent observations from a single specimen only and are not statistically significant.

To present the inter-group differences in tissue injury more intuitively, the above pathological observations were semi-quantitatively scored; the results are presented in Table 1 and Figure 7 and Figure 8. Group C had a total score of 0, indicating entirely normal intestinal architecture; group H had a total score of 6, with damage mainly affecting the villi and the lamina propria–submucosa; group K had a total score of 11, exhibiting moderate to severe injury across all four evaluated dimensions; and group R had a total score of 4, markedly lower than that of group K, indicating an alleviating trend. The scoring trend was consistent with the gene expression changes and morphological descriptions (Table 1 and Figure 7 and Figure 8); however, with *n* = 1 per group these scores are descriptive only and were not subjected to statistical analysis.

Although these histopathological observations are preliminary, they show trends consistent with the changes in gene expression: suppression of *sirt1* function (group K) was accompanied by more pronounced tissue injury, whereas resveratrol pretreatment (group R) exerted a degree of protection.

## 4. Discussion

### 4.1. Tissue Distribution of sirt1 and Its Time-Course Expression Pattern Under Hypoxia

Bioinformatic analyses showed that yellow catfish Sirt1 contains a conserved SIR2 catalytic domain and shares high sequence identity with Sirt1 proteins of other vertebrates, with the highest identity (80.36%) observed with channel catfish (order Siluriformes) and 54.92% with human Sirt1. Phylogenetic analysis confirmed the closest relationship was with channel catfish Sirt1 (bootstrap 100%). This evolutionary conservation provides a molecular basis for inferring conserved metabolic regulatory functions of Sirt1 in the hypoxic response of yellow catfish.

Under normoxic conditions, *sirt1* mRNA was most highly expressed in the brain of yellow catfish, followed by the intestine—the intestinal level was significantly higher than that of the heart and kidney (*p* < 0.05)—while muscle showed the lowest expression. This pattern is consistent with the preferential expression of Sirt1, an NAD^+^-dependent deacetylase, in metabolically active tissues, and resembles distributions reported in zebrafish [23], largemouth bass [25], and gilthead seabream [26,49]. The high basal expression in the brain likely reflects its extreme oxygen demand and the role of Sirt1 in neuronal energy metabolism through NAD^+^ sensing [12,16]. The relatively high intestinal expression is particularly noteworthy: as the central organ of digestion and absorption, the intestine exhibits high metabolic activity [9] and has been identified as a critical target of hypoxic stress in yellow catfish. Wang et al. [3] reported marked oxidative stress in this tissue after acute hypoxia–reoxygenation, and a recent multi-omics study revealed extensive intestinal damage under acute hypoxia at both the transcriptional and metabolic levels [10]. Together, these findings support a central role for the intestine in Sirt1-mediated hypoxic regulation.

Combining the results of the functional validation experiment (6 h) with those of the time-course experiment (12 h), the present study revealed that *sirt1* transcription was decreased during the early phase of hypoxia (6 h) and then significantly upregulated at 12 h. Although the two time points derive from independent experiments and direct comparison has methodological limitations, the data collectively indicate a time-dependent biphasic response. Early downregulation is consistent with the mechanism described by Lim et al. [48], in which hypoxia-induced decreases in the NAD^+^/NADH ratio activate CtBP, which then represses *sirt1* transcription. The subsequent upregulation likely reflects adaptive compensation, in which AMPK activation partially restores NAD^+^ availability and relieves CtBP-mediated repression [17], while HIF-1α-dependent transcription via hypoxia response elements in the *sirt1* promoter further enhances expression [49]—potentially functioning as a delayed negative-feedback loop attenuating excessive HIF-1α activity. This “first decreasing then increasing” pattern thus likely reflects a temporal transition from acute metabolic crisis to adaptive compensation signaling.

Among the other sirtuin family members examined in parallel, *sirt3* and *sirt4* showed sustained downregulation, whereas *sirt6* exhibited delayed upregulation, peaking at 72 h. These patterns are temporally complementary to the early response of *sirt1* and may represent a long-term adaptive strategy of the organism to sustained hypoxic stress [30].

### 4.2. Regulatory Role of sirt1 in the Acute Hypoxic Response

To investigate the function of *sirt1* in hypoxia, the groups H, K, and R were compared. Group R underwent a one-week recovery after siRNA injection before hypoxic exposure, whereas group K was exposed 24 h after the final injection. Because siRNA-mediated silencing wanes over time, transcriptional suppression of *sirt1* at the point of hypoxic challenge was likely weaker in group R than in group K, which must be considered when interpreting between-group comparisons.

At the level of energy metabolism, acute hypoxia (6 h) markedly activated AMPK signaling: *ampkα* expression in group H was upregulated approximately 1.6-fold. AMPK is a conserved cellular energy sensor activated when the AMP/ATP ratio rises under impaired mitochondrial respiration [50,51], and is a likely mediator of the metabolic reprogramming previously documented in yellow catfish under hypoxia [3,8,10]. AMPK and Sirt1 are linked by a positive-feedback relationship: AMPK elevates intracellular NAD^+^ to activate Sirt1, while Sirt1 deacetylates liver kinase B1 (LKB1) to enhance AMPK activity [17,52]. In group K, *sirt1* RNAi reduced *ampkα* expression to the control level, suggesting that this AMPK–Sirt1 axis is coordinately regulated at the transcriptional level.

This metabolic activation was accompanied by altered regulation of HIF-1α target genes. *pdk1*, a direct HIF-1α target [53], was upregulated approximately 3.8-fold in group K. PDK1 phosphorylates and inactivates pyruvate dehydrogenase, redirecting metabolism from oxidative phosphorylation toward glycolysis [54]. Previous studies indicate that Sirt1 can deacetylate HIF-1α at Lys674 and thereby inhibit the transcription of HIF-1 target genes [48], although other studies report that Sirt1-mediated deacetylation can stabilize the HIF-1α protein and enhance its transcriptional activity [18,20]. In the present study, the pronounced upregulation of *pdk1* in group K, together with the aggravated tissue injury, is more consistent with a negative regulatory role of Sirt1 on HIF-1α target genes: loss of Sirt1 releases inhibition of HIF-1α activity, leading to abnormally high *pdk1* expression and excessive glycolytic shift, which may increase the risk of cellular damage. In contrast, *pgk1* was downregulated at 6 h, with group H expression at approximately 0.2-fold that of the control, possibly reflecting a transitional state in which the early hypoxic metabolic switch has not yet been fully established. Whether reduced PGK1, which can translocate to the mitochondria to coordinate glycolysis with the tricarboxylic acid (TCA) cycle [55], contributes to the abnormally high *pdk1* expression in group K requires further investigation.

Parallel changes were observed in signal-transduction pathways. *stat3* was significantly downregulated in all hypoxic treatment groups. STAT3 can form a complex with HIF-1α to cooperatively regulate hypoxia-responsive genes [56], and Sirt1 can modulate STAT3 activity through deacetylation [57,58]. Partial restoration of *stat3* in group R suggests that recovery of Sirt1 function may alleviate this suppression, although the time-dependent decay of siRNA silencing in group R cannot be excluded as a contributing factor. *mapk1* was downregulated in group H but upregulated in groups K and R, showing a pattern opposite to that produced by hypoxia alone and an inverse relationship with the *sirt1* expression level. Under hypoxia alone (group H), this downregulation may reflect HIF-1α-mediated suppression of mitogen-activated protein kinase (MAPK)/extracellular signal-regulated kinase (ERK) signaling as part of a shift away from proliferative responses toward survival responses. Upon *sirt1* knockdown in group K, the loss of Sirt1-mediated deacetylation of upstream regulators may relieve negative feedback within the MAPK/ERK cascade [57,58], releasing this suppression and resulting in *mapk1* upregulation. Because MAPK1 participates in cell survival, proliferative and pro-inflammatory signaling cascades, the elevated *mapk1* transcript level consequent to *sirt1* suppression is consistent with the more pronounced intestinal epithelial injury and inflammatory infiltration observed histopathologically in group K (Section 3.6). In group R, upregulation despite partial Sirt1 recovery may reflect the pleiotropic effects of resveratrol on MAPK signaling independent of Sirt1 [59].

**Figure 9:**
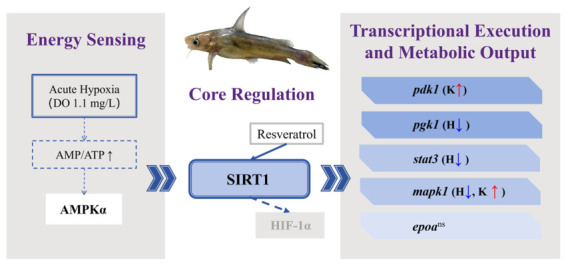
Working transcriptional response network involving Sirt1 under acute hypoxic response in the intestine of yellow catfish. Schematic diagram of the *sirt1* regulatory network constructed on the basis of the present experimental results and the existing literature. Solid arrows indicate regulatory relationships supported by the present data, whereas dashed arrows represent regulatory relationships inferred from the literature. Red arrows denote upregulation and blue arrows denote downregulation.

On the basis of these results and the existing literature, a working transcriptional response network involving *sirt1* in yellow catfish intestine under acute hypoxic stress is proposed (Figure 9).

At the energy-sensing level, hypoxia-induced elevation of the AMP/ATP ratio activates AMPK [50], which engages the AMPK–Sirt1 positive-feedback loop [17,52]; the upregulation of *ampkα* in group H and its decline in group K support this axis. At the central regulatory level, Sirt1 modulates HIF-1α stability and activity through deacetylation [18,19,47]; loss of Sirt1 disrupts hypoxic signaling, leading to aberrant downstream gene expression and tissue damage. At the transcriptional execution level, stabilized HIF-1α binds hypoxia response elements together with HIF-1β, while STAT3 acts as a co-transcription factor [56]. At the effector level, *pdk1*-mediated metabolic switching [53,54] and *pgk1*-mediated glycolytic ATP production [55] mediate acute adaptation, while *epoa*-mediated enhancement of oxygen-carrying capacity contributes over longer timescales [60]. The lack of significant *epoa* response in any treatment group at 6 h may be attributable to two factors: first, the relatively short hypoxic exposure may have been insufficient to sustain the HIF-1α transcriptional activity required to drive a robust erythropoietic response [60]; second, erythropoietin production in teleosts occurs primarily in the kidney and liver rather than the intestine [60], so intestinal *epoa* may be intrinsically less responsive to short-term acute hypoxia regardless of *sirt1* status. The absence of a significant *epoa* change in the intestine at this time point is therefore interpreted as a tissue- and time-point-specific finding rather than evidence against a broader transcriptional response of *sirt1* in the intestinal hypoxic response, and does not negate the systemic role of *epoa*.

### 4.3. Alleviating Effects of Resveratrol on Hypoxia-Induced Intestinal Injury

Drawing on mammalian studies, the tissue-protective effects of Sirt1 are thought to involve several mechanisms: Sirt1 can deacetylate the nuclear factor κB (NF-κB) p65 subunit to inhibit the expression of pro-inflammatory cytokines [61]; it can deacetylate p53 and members of the FOXO family of transcription factors to regulate apoptotic programs [62]; and it can activate the expression of antioxidant enzymes to mitigate oxidative stress [63]. The high basal expression of *sirt1* in the intestine, together with the present histopathological findings, is consistent with a transcriptional response pattern of *sirt1* associated with the maintenance of intestinal homeostasis under hypoxic conditions. Because only one sample per group was available for histopathological analysis, these conclusions require validation in future studies with larger sample sizes.

Resveratrol is an effective Sirt1 activator [59]. In the present study, *sirt1* mRNA in group R did not differ significantly from group H, yet downstream genes showed partial recovery: *pgk1* and *stat3* levels rebounded relative to group K, and intestinal morphological injury was alleviated. These observations are consistent with the established mechanism in which resveratrol acts primarily by enhancing the deacetylase activity of residual Sirt1 protein rather than by increasing its transcription [59], thereby partially compensating for the functional deficit caused by reduced *sirt1* expression. Notably, resveratrol is biologically pleiotropic and exerts direct antioxidant and anti-inflammatory effects independent of Sirt1 [59], so the protection observed in group R likely reflects combined mechanisms.

In aquaculture, dietary resveratrol has been shown to improve growth performance and antioxidant capacity in cultured fish [64,65]. Yellow catfish—a commercially important species frequently exposed to fluctuating dissolved oxygen in pond and raceway systems [1,2]—are routinely cultured under intensive conditions that predispose them to episodic hypoxia [3]. The present results therefore provide preliminary evidence supporting resveratrol as a candidate functional feed additive for hypoxia resistance in this species, although the optimal dose, bioavailability under practical feeding conditions [35], and the relative contributions of Sirt1-dependent versus Sirt1-independent mechanisms remain to be defined.

### 4.4. Limitations and Future Directions

Several limitations of the present study should be acknowledged.

First, many molecular interactions in the proposed regulatory model are derived primarily from mammalian studies, and their precise mechanisms in fish remain to be validated. The present work measured gene expression mainly at the transcriptional level without directly quantifying Sirt1 protein abundance or enzymatic activity; the inference that Sirt1 regulates downstream targets through HIF-1α deacetylation therefore remains a hypothesis requiring confirmation by Western blotting, co-immunoprecipitation, and deacetylation assays. Regulation at additional levels—post-translational modifications, microRNA control, and epigenetic mechanisms—may also contribute and warrant investigation.

Second, several physiologically informative endpoints were not measured. The intracellular NAD^+^/NADH ratio, AMPK phosphorylation status, HIF-1α protein levels, and apoptosis-related indicators (e.g., caspase-3 activity and Bcl-2/Bax ratio) would clarify the mechanisms underlying both the AMPK–Sirt1 axis and tissue injury, and should be included in future experiments. Dedicated assays of MAPK phosphorylation are also needed to clarify Sirt1’s role in this pathway in yellow catfish intestine. In addition, although the fish were continuously monitored throughout the hypoxic exposure and deeply anesthetized before sampling in accordance with the approved IACUC protocol, the present study did not include quantitative welfare-related stress indicators (e.g., plasma cortisol, behavioral scoring, and survival monitoring at defined humane endpoints) or product-quality traits (e.g., muscle pH, water-holding capacity, texture, and nutritional composition); future studies in this species should incorporate such endpoints to relate the intestinal transcriptional responses characterized here to whole-animal welfare and to aquaculture-relevant product traits.

Third, the biphasic regulation of *sirt1* was inferred from two independent experiments, and continuous time-course experiments with simultaneous measurement of NAD^+^/NADH ratios, AMPK phosphorylation, and HIF-1α protein are needed to rigorously validate this dynamic. In addition, histopathological analysis was based on a single representative specimen per group as a supplementary semi-quantitative reference; although the directional trends were consistent with the gene expression data, future studies with adequate sample sizes for quantitative scoring are needed to confirm these structural observations.

Fourth, the hypoxic-response experiments focused on intestinal tissue. Given the high basal *sirt1* expression in the brain and heart, the hypoxic response characteristics of *sirt1* in these tissues warrant further investigation. Sampling the kidney and liver, or extending the exposure duration, would also be more appropriate for capturing EPO-mediated systemic adaptation.

Future research should advance along several directions: (i) establishing *sirt1* gene-edited yellow catfish lines for definitive genetic validation; (ii) performing hypoxia–reoxygenation cycle experiments to evaluate recovery dynamics; and (iii) systematically optimizing dietary resveratrol supplementation, including dose–response and bioavailability studies, to translate the present mechanistic findings into practical aquaculture applications.

## 5. Conclusions

The present study characterized the sequence features of the *sirt1* gene in yellow catfish, examined its tissue distribution and dynamic transcriptional response under acute hypoxia, and assessed the functional consequences of *sirt1* knockdown and resveratrol pretreatment on downstream gene expression and intestinal histology. First, bioinformatic analysis demonstrated that the SIR2 catalytic domain of the yellow catfish Sirt1 protein is highly conserved among vertebrates. Second, *sirt1* was preferentially and highly expressed in metabolically active tissues such as the brain and intestine, and was the most hypoxia-responsive member of the *sirt* family in yellow catfish, being significantly upregulated to approximately 4.5-fold that of the control at 12 h of hypoxia; together with the downregulation observed at 6 h of acute hypoxia, this points to a time-dependent dynamic transcriptional response of *sirt1* during acute hypoxia. Finally, functional validation experiments indicated that *sirt1* shows a transcriptional pattern consistent with a central role in the hypoxic response: its downregulation led to attenuated *ampkα* transcriptional upregulation, pronounced compensatory upregulation of *pdk1*, and aggravated intestinal injury, whereas resveratrol partially restored downstream gene expression and alleviated intestinal damage. Together, these findings indicate that *sirt1* is an transcriptionally responsive gene in the response of yellow catfish to hypoxic stress and provide a basis for further investigation into hypoxia adaptation in yellow catfish.

## Figures and Tables

**Table 1:** Semi-quantitative histopathological scores of midgut tissues in each treatment group of yellow catfish. The scoring criteria were adapted from the semi-quantitative histopathological scoring scheme for fish established by Baums et al. [42], with modifications based on the histological characteristics of the yellow catfish intestine. Each item was scored on a 0–3 scale, with 0 = normal, 1 = mild injury, 2 = moderate injury, and 3 = severe injury, with a total score ranging from 0 to 12. Scoring was performed twice independently by an assessor experienced in fish histopathology, and the mean was taken. With *n* = 1 per group, these scores do not support statistical analysis and serve only as a supplementary semi-quantitative reference to the morphological description.

Scoring Item and Score	Group C	Group H	Group K	Group R
Epithelial Integrity	0	1	3	1
Villus Structure	0	2	3	1
Lamina Propria–Submucosa Inflammation	0	2	3	1
Muscle Layer Structure	0	1	2	1
Total Score	0	6	11	4

## Data Availability

Data will be made available on request.

## Supplementary Materials

The following supporting information can be downloaded at: https://www.mdpi.com/article/10.3390/ani16111679/s1. Figure S1: Partial multiple sequence alignment of yellow catfish Sirt1 against homologous proteins from nine representative vertebrate species. Table S1: Semi-quantitative histopathological scoring criteria for intestinal tissue. Table S2: Primer sequence list.

## Abbreviations

The following abbreviations are used in this manuscript:

AMPK	AMP-activated protein kinase
H&E	Hematoxylin and eosin
HIF-1α	Hypoxia-inducible factor 1α
JTT	Jones–Taylor–Thornton
LSD	Least significant difference
MS-222	Ethyl 3-aminobenzoate methanesulfonate
NAD^+^	Nicotinamide adenine dinucleotide
NJ	Neighbor-Joining
Res	Resveratrol
RNAi	RNA interference
ROS	Reactive oxygen species
siRNA	Small interfering RNA
