# Supplementary material for: Expression and Transcriptional Response of sirt1 in Yellow Catfish (Pelteobagrus fulvidraco) Under Acute Hypoxia Stress
Source: Animals (Basel). 2026 May 30;16(11):1679. doi: 10.3390/ani16111679 (PMC13255791; doi:10.3390/ani16111679)
Supplement: Supplementary file 1 [file animals-16-01679-s001.zip › animals-4337067-supplementary.pdf]

Supplementary Materials

Table S1 Semi-quantitative Histopathological Scoring Criteria for Intestinal Tissue

Scoring Item and Score	Scoring Criteria
Epithelial Integrity	
Score 0	Epithelial cells are arranged in an orderly columnar pattern with tight intercellular junctions and no desquamation.
Score 1	Focal loosening or mild flattening of epithelial cell arrangement, with occasional single-cell desquamation.
Score 2	Marked thinning of the epithelial layer with disorganized cell arrangement and multiple foci of epithelial shedding or disruption.
Score 3	Extensive epithelial desquamation or loss with exposed basement membrane and complete loss of columnar architecture.
Villus Structure	
Score 0	Villi are morphologically intact, slender, regularly arranged, and of uniform height.
Score 1	Mild edema or blunting at the villus tips, with slightly reduced length; overall architecture largely preserved.
Score 2	Marked villus atrophy, shortening, or fusion, with partial villus disruption visible.
Score 3	Severe villus atrophy or extensive necrotic shedding, with severely disordered luminal surface architecture.
Lamina Propria – Submucosa Inflammation	
Score 0	Normal cell density in the lamina propria/submucosa, with no obvious inflammatory cell infiltration.
Score 1	Sparse scattered inflammatory cell infiltration, focally distributed.
Score 2	Moderate inflammatory cell infiltration, diffusely distributed across multiple regions, with mild tissue edema.
Score 3	Dense and abundant inflammatory cell infiltration with marked thickening or edema of the lamina propria.
Muscle Layer Structure	
Score 0	The circular and longitudinal muscle layers are regularly arranged, with orderly fiber orientation and clear laminar organization.
Score 1	Mildly loosened muscle fiber arrangement, with occasional widening of interfibrillar spaces.
Score 2	Disorganized muscle fiber arrangement with blurred lamination and focal disruption or edema.
Score 3	Severely disordered or disintegrated muscle layer structure with

complete loss of fiber arrangement.

Table S2 Primer Sequence List

Primers name	Primer sequence (5'-3')	Product length (bp)	GenBank accession number
β-actin-F	GGATTCGCTGGAGATGATG	221	XM_027148463.2
β-actin-R	TCGTTGTAGAAGGTGTGATG		
sirt1-F	GCGTTAGAAACGGGTTATTTGG	137	XM_027165852.2
sirt1-R	CGGTAAGACCCAAAACGTGG		
sirt2-F	CTGCAGGTTCAACCCTTTGC	166	XM_027162160.2
sirt2-R	GTGCAACATCCCTGTAGGCT		
sirt3-F	TGTGCGAGGTTCTGTGTCTC	169	XM_027153054.2
sirt3-R	AGCCATAAGAGTTTCCAGCTCA		
sirt4-F	GAAGCGTTTTGCTGCACTGA	178	XM_047813469.1
sirt4-R	TGCACGGTCTACCACATCAC		
sirt5-F	GTCAAATTCACCGGGTTGGC	184	XM_027179597.1
sirt5-R	AATCCTGAGCCTGCCACTTC		
sirt6-F	CCTCATCAGCCAAAACGTGG	111	XM_027150779.2
sirt6-R	TTGCCGCACTTCTCACATTC		
sirt7-F	CAGGGGCATCAAGACACAGT	162	XM_047803095.1
sirt7-R	GTAAACGGCAACATGGCGAG		
ampkα-F	TTACAGACGATTCGGCCAGC	118	XM_027165473.2
ampkα-R	GTTCTGGATCTCACGGCGAA		
mapk1-F	GATGAATGCTGCTCGGGGAT	111	XM_027162841.2
mapk1-R	CAGTGAAAGCGACGGAGACT		
pgk1-F	AGGCTGTAAGCAGAGCGAAG	127	XM_027177360.2
pgk1-R	ATGCAACCGCTTTTGGTCAC		
stat3-F	GTCACTCCGTGTCCTTCCAT	149	XM_027143685.2
stat3-R	GCTTCAGTCTCAGGGTGCAT		
pdk1-F	CTGACCCTGTAACCAGCCAG	117	XM_027164436.2
pdk1-R	CCTGCACCTTCCCTCCAAAT		
epoa-F	ATCTGCGACCTGCGTGTTCT	179	XM_027164944.2
epoa-R	GTCTGGACCTCTTGAGCCTGTT		
si*sirt1*-1-S	CGGUCGACGUUAUAGCUAACU	-	XM_027165852.2
si*sirt1*-1-AS	UUAGCUAUAACGUCGACCGAU		
si*sirt1*-2-S	CAGACAGUAUAAUGAAGAAUC	-	XM_027165852.2
si*sirt1*-2-AS	UUCUUCAUUAUACUGUCUGUG		
si*sirt1*-3-S	GGAAUGUAACAAAGUUCAAAC	-	XM_027165852.2
si*sirt1*-3-AS	UUGAACUUUGUUACAUUCCUG		
si_nc-S	ACGUGACACGUUCGGAGAA/dT//dT/	-	XM_027165852.2
si_nc-AS	UUCUCCGAACGUGUCACGU/dT//dT/		

Fig. S1. Partial multiple sequence alignment of yellow catfish Sirt1 against homologous proteins from nine representative vertebrate species.

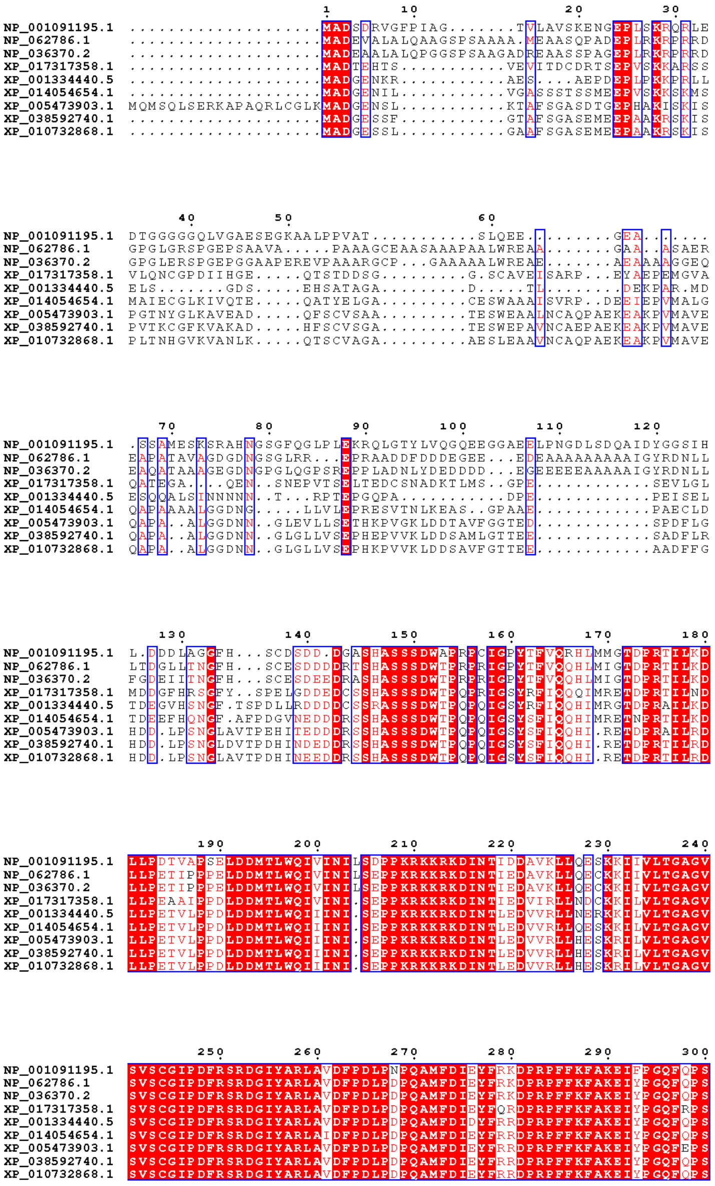

```
                       310       320       330       340       350       360
NP_001091195.1  LCHRFIAMLDKEGKLLRNYTQNIDTLEQVAGIEKIIQCHGSFAEASCLVCKYKVDCEAVR
NP_062786.1     LCHKFIALSDKEGKLLRNYTQNIDTLEQVAGIQRILQCHGSFATASCLICKYKVDCEAVR
NP_036370.2     LCHKFIALSDKEGKLLRNYTQNIDTLEQVAGIQRIIQCHGSFATASCLICKYKVDCEAVR
XP_017317358.1  LCHRFIAMLDKKEKILRNYTQNIDTLEQVAGVQRVMQCHGSFATASCLVCKHKVDCDAIR
XP_001334440.5  PCHRFISMLDKKGRLLRNYTQNIDTLEQVAGIQKIIQCHGSFATASCLICKHKVDCEAIR
XP_014054654.1  PCHKFISMLDKQGKLLRNYTQNIDTLEQVAGVQRIIQCHGSFATASCLVCKHKVDCEAVR
XP_005473903.1  PCHRFISMLDKQRKLLRNYTQNIDTLEQVAGVQRIIQCHGSFATASCLVCKHKVDCEAIR
XP_038592740.1  PCHKFISMLDKKGKLLRNYTQNIDTLEQVAGVQRIIQCHGSFATASCLVCKHKVDCEAVR
XP_010732868.1  PCHKFISMLDKQGKLLRNYTQNIDTLEQVAGVQRIIQCHGSFATASCLVCKHKVDCEAIR
```

```
                       370       380       390       400       410       420
NP_001091195.1  EDIFNQIVPRCPQCSPDEPLAIMKPDIVFFGENLPEQFHRAMKYDKNEVDLLIVIGSSLK
NP_062786.1     GDIFNQVVPRCPRCPADEPLAIMKPEIVFFGENLPEQFHRAMKYDKDEVDLLIVIGSSLK
NP_036370.2     GDIFNQVVPRCPRCPADEPLAIMKPEIVFFGENLPEQFHRAMKYDKDEVDLLIVIGSSLK
XP_017317358.1  EDIFRQVVPHCPRCH.NIPFAIMKPDIVFFGENLPEAFHRAMKQDKDEVDLLIVIGSSLK
XP_001334440.5  EDIFNQVVPHCPRCPSDVPYAIMKPDIVFFGENLPEFFHRAMKQDKDEVDLLIVIGSSLK
XP_014054654.1  EDIFYQVVPHCPRCP.DIPMAIMKPDIVFFGENLPELFHRAMRQDKDEVDLLIVIGSSLK
XP_005473903.1  EDIFNQVVPHCPRCP.DIPLAIMKPDIVFFGENLPEMFHRAMKQDKDEVDLLIVIGSSLK
XP_038592740.1  EDIFNQVVPHCPRCP.DIPLAIMKPDIVFFGENLPEMFHRAMKQDKDEVDLLIVIGSSLK
XP_010732868.1  EDIFNQVVPHCPRCP.DIPLAIMKPDIVFFGENLPEMFHRAMKQDKDEVDLLIVIGSSLK
```

```
                              430       440       450       460       470
NP_001091195.1  VRPVALIPS.......SIPHEVPQILINREPLPHLHFDIELLGDCDVIINELCQRLDGKY
NP_062786.1     VRPVALIPS.......SIPHEVPQILINREPLPHLHFDVELLGDCDVIINELCHRLGGEY
NP_036370.2     VRPVALIPS.......SIPHEVPQILINREPLPHLHFDVELLGDCDVIINELCHRLGGEY
XP_017317358.1  VRPVALIPSMSQLIDGSVPPEVPQVLINREPLLHLNFDVELLGDCDVIVNELCHRLGGDF
XP_001334440.5  VRPVALIPS.......SIPHDVPQVLINREPLPHLNFDVELLGDCDVIVNELCHRLNGDF
XP_014054654.1  VRPVALIPT.......SIPHDVPQVLINREQLPHLNFDVELLGDCDVIVNELCHRLGGDF
XP_005473903.1  VRPVALIPN.......SIPHEVPQVLINREQLPHLNFDVELLGDCDVIINELCHRLGGDF
XP_038592740.1  VRPVALIPN.......SIPHEVPQVLINREQLPHLNFDVELLGDCDVIVNELCHRLGGDF
XP_010732868.1  VRPVALIPN.......SIPHEVPQVLINREQLPHLNFDVELLGDCDVIVNELCHRLGGDF
```

```
                    480       490       500       510
NP_001091195.1  SQLCTNFLKLSQITEMPPRIHKGFLTSPETIPSTDLNTGQ.SVEL.....KR........
NP_062786.1     AKLCCNPVKLSEITEKPPRPQKELVHLSELPPT.PLHISEDSSSP.....ER..TVPQDS
NP_036370.2     AKLCCNPVKLSEITEKPPRTQKELAYLSELPPT.PLHVSEDSSSP.....ER..TSPPDS
XP_017317358.1  EQLCYNSSRLSEITDTPPPLPTTLPEQACAKVRTPEVQNTG.....QFFTEQD.......
XP_001334440.5  QQLCYNSSRLSEITEKPAAPEHTENTSADHSHADAEHIENTSADRDDADAEHIENTSADH
XP_014054654.1  EQLCYNSLRLNEITENPPRLATSAEQPSCKA......LSTH...SKPTEKEQK.HRHTDS
XP_005473903.1  EQLCYNTVRLTEITEKPPRLPE...QPPSEA......LSAS...SDATQEEQK.QHTTDS
XP_038592740.1  EQLCYNTVRLNEITEKPPRLPE...QPPSEA......LLPS...SDAAQEEQK.QYKTDS
XP_010732868.1  EQLCYNTVRLTEITEKPPRLPE...QLPSDA......LPAS...SDAAQEELT.QHSTDS
```

```
                                520
NP_001091195.1  ..................ELHE.....................TDLLSA...........
NP_062786.1     SV.....IAT.LVDQ..ATNNN.....................VNDLEV...........
NP_036370.2     SV.....IVT.LLDQ..AAKSN.....................DDLDVS...........
XP_017317358.1  .....ENIAE.................................TSNTVTVIDSCPNAQFS
XP_001334440.5  SHADAEHIENTSADRDDADAEHIENTSADHSHADAEHIENTSADRDDAEHTENTPTDHAN
XP_014054654.1  SVSASEE..........TNTHMVSD................TPVR.DRIPTEPCPTAQNV
XP_005473903.1  VNMPSEE..........TESLNVTE................TADN.NVTPLEPCPNA...
XP_038592740.1  VTKPPEE..........TESHSVTE................TAGN.NVTPPEPCPNA...
XP_010732868.1  VSKPSEE..........RESPNVTE................TAGN.NVTPPEPCPNA...
```

```
                  530                                540       550
NP_001091195.1  ....SNTACSLEKP.......................KEASKLPHSCTDENLEVFK....
NP_062786.1     ....SESSCVEEKP.......................QEVQTSR......NVENIN....
NP_036370.2     ....ESKGCMEEKP.......................QEVQTSR......NVESIA...E
XP_017317358.1  IGGGLNTSCTTEEVRSSPELENRSSSPKPLTQENDGCP..NAQPSPCRDRSNQ.......
XP_001334440.5  A.....................EHTEHTSAGHVNAEHIEHMSTDHANAKDDQSSLSVNEE
XP_014054654.1  T..............DTPEVDGVPPEPCPKGQIVQDTPEVVAQPEPCP............
XP_005473903.1  ............................................................
XP_038592740.1  ............................................................
XP_010732868.1  ............................................................
```

```
                  560                 570       580       590       600
NP_001091195.1    EVNTQSLKG..A................SQTETQPSNEKDQETTDKDTDIDSTKDLENKY
NP_062786.1       .VENPDFKA..V................GSS....TADKNER...TSVAETVRKCWPNRL
NP_036370.2       QMENPDLKN..V................GSS....TGEKNER...TSVAGTVRKCWPNRV
XP_017317358.1    ..NAP.........VEAVGHRLNT....QQEREEMLDMQTGEKHTNQRNDIRKQCWLKQI
XP_001334440.5    ELASPAAETHALDSTEISAHTERSKEADAVNTDDAACVKDEENTDRLRVEMRRRCWRSRI
XP_014054654.1    KAPSPNEER..MEYSVSPS............TDTRREEGSGLNDQHINLEVRGLCWMSRI
XP_005473903.1    ..QCPTEST..ESPE................KGAPKEEAAERKSQTSNLEFRRRCWMSRV
XP_038592740.1    ..QCPSEET..AEPSELSA............EDTPKEEAAELKSQTSNLEFRRRCWMSRI
XP_010732868.1    ..QCPSEET..AEPSELSA............EDTPKEEAAEVKSQTSNLELRRRCWMSRI

                        610       620       630        640       650
NP_001091195.1    TKEQISKRLDSTQFLFLAPNRYIFHGAEVFSDSEEE.LTSSSCGTNSDSESLLSPSLHEP
NP_062786.1       AKEQISKRLEGNQYLFVPPNRYIFHGAEVYSDSEDDVLSSSSCGSNSDSGTCQSPSLEEP
NP_036370.2       AKEQISRRLDGNQYLFLPPNRYIFHGAEVYSDSEDDVLSSSSCGSNSDSGTCQSPSLEEP
XP_017317358.1    CRNPISNRLGTSQYLFQVPNRYIFHGAEVYSCSEDE..TSSSS..DNEGSCCSGSGLEEQ
XP_001334440.5    CQSPISKRLGASQYLFQAPNRYVFHGAEVYSSSEDE..SSSSCSSESDGSFQHED.....
XP_014054654.1    CRSPISKRLETSQYLFRAPNHYIFHGAEVYSNSEDE..TSSSCESEDSSLE.....MEED
XP_005473903.1    NRTPISKRLEAGQYLFQPPNHYIFHGAEVYSDSEDE..TSSSCGSDSEDSECCAEGEEDD
XP_038592740.1    NRSPISKRLETGQYLFQAPNHYIFHGAEVYSDSEDE..TSSSCGSDSDESECSADGVEED
XP_010732868.1    NRSPISKRLETGQYLFQAPNHYVFHGAEVYSDSEDE..TSSSCGSDSDESECSVEGGEED

                 660       670       680          690       700
NP_001091195.1    IEEESETEECFHAKYENETDTDNRAD...LEKEP.ERVVLYESDD.......L...LGNG
NP_062786.1       LEDESEIEEFYNGLEDD.TERPECAGGSGFGADGGDQEVVNEAIA.......T...RQEL
NP_036370.2       MEDESEIEEFYNGLEDE.PDVPERAGGAGFGTDGDDQEAINEAIS.......V...KQEV
XP_017317358.1    ESYA....EEECTPGDCPS.....PGEFGVG.ETVN...ENKCMSSI.HTDSIKGNLKTM
XP_001334440.5    SEVE....ENGAAMTDKET......DTETVQ.DSEHRRLQTHCTQHTQ............
XP_014054654.1    SDAE....DEQSTPTE..E......VEGCLI.DTVTRKKACEGTLSVKQTDNTETNVQNT
XP_005473903.1    SEPE....DADIVPAV..D......GETCLK.DIVQHTVATEATSSVQT.DNNSEKTEST
XP_038592740.1    SEPE....DAGVL.AA..D......GETCLR.DTIQHTLANEATSSAPT.DNISEKTQST
XP_010732868.1    SEPE....EAATL.AV..E......GETCLR.DTIQHTLANEATSSVQT.DNTSEKTQST

                     710
NP_001091195.1    TTMNL.....
NP_062786.1       TDVNYPSDKS
NP_036370.2       TDMNYPSNKS
XP_017317358.1    TDL.......
XP_001334440.5    ..........
XP_014054654.1    TDL.......
XP_005473903.1    THL.......
XP_038592740.1    THL.......
XP_010732868.1    THL.......
```
